# Amniotic-Umbilical-to-Cerebral Ratio (AUCR) as a diagnostic tool for predicting adverse perinatal outcome in pregnancies complicated by preeclampsia

**DOI:** 10.1007/s00404-026-08531-y

**Published:** 2026-09-21

**Authors:** L. M. Müller, K. Oelmeier, R. Schmitz, M. Möllers, K. Oberste, D. Willy

**Affiliations:** 1 Department of Gynaecology and Obstetrics, University Hospital Münster, Albert-Schweitzer-Campus 1, 48149 Münster, Germany; 2 Department of Gynaecology and Obstetrics, Florence-Nightingale-Krankenhaus, 40489 Düsseldorf, Germany

**Keywords:** Preeclampsia, Doppler parameters, Adverse perinatal outcome, AUCR, Foetal growth restriction, Placental insufficiency

## Abstract

**Purpose:**

To compare the ability of different Doppler parameters in predicting composite adverse perinatal outcomes in women with preeclampsia. We especially aimed to analyse a possible future role of novel Doppler ratios, such as the Amniotic-Umbilical-to-Cerebral Ratio (AUCR), in a high-risk cohort, such as women suffering from preeclampsia.

**Methods:**

A retrospective cohort study was conducted, analysing data from 151 pregnant women, consisting of 54 with preeclampsia and 97 healthy controls. Various Doppler parameters, including umbilical artery Doppler, middle cerebral artery, as well as computed ratios, such as the cerebroplacental ratio (CPR) and AUCR, were evaluated for their predictive value regarding composite adverse perinatal outcomes (CAPOs). Receiver Operating Characteristic (ROC) analyses were performed to assess the diagnostic accuracy of each parameter, and the area under the curve (AUC) was calculated.

**Results:**

Our results showed that AUCR was the strongest predictor of composite adverse perinatal outcomes among women with preeclampsia. In comparison, AUCR showed low predictive accuracy in the low-risk cohort of healthy controls. The AUC ROC values for each Doppler parameter were calculated, with AUCR yielding an AUC value of 0.87, indicating good predictive performance.

**Conclusion:**

This study suggests that AUCR is a valuable tool for predicting composite adverse perinatal outcomes in women with preeclampsia. The findings of this investigation may lead to further studies on AUCR, but require prospective validation.

## **What does this study add to the clinical work**

**Table:** 

The AUCR may serve as a valuable Doppler index for identifying preeclamptic pregnancies at increased risk of adverse perinatal outcomes; prospective validation is required before routine clinical use.	

## Introduction

Preeclampsia is a leading cause of maternal and perinatal mortality worldwide, affecting 2–5% of pregnancies [1]. Infants born to mothers with preeclampsia are more often born prematurely, have a lower birth weight and face a higher risk of neonatal morbidity and mortality.

The monitoring of foetal well-being during pregnancy is crucial, especially in pregnancies at an increased risk for perinatal complications. Various methods are employed to assess foetal status, including ultrasonographic assessment of foetal growth, cardiotocography (CTG), and Doppler ultrasound.

The International Society for the Study of Hypertension in Pregnancy recommends applying the ISUOG foetal-growth-restriction surveillance protocol to all pregnancies affected by preeclampsia, because both share underlying mechanisms of placental dysfunction and carry an increased risk of perinatal mortality [2]. Assessment of foetal growth in combination with Doppler ultrasound makes it possible to identify and distinguish between foetuses that are small for gestational age (SGA) and those with foetal-growth-restriction (FGR).

FGR is generally defined as a foetus failing to reach its genetically predetermined growth potential, whereas SGA foetuses fall below a predefined threshold for their gestational age despite not necessarily being at increased risk of adverse perinatal outcomes [3].

Management of FGR faces health care providers with the challenge of optimal timing of delivery avoiding unnecessary risks and mortality caused by prematurity versus mortality caused by antenatal hypoxemia. The TRUFFLE study has proven the superiority of combining computerised CTG and measurement of the ductus venosus as a trigger for delivery in early-onset FGR [4].

Numerous studies have explored the predictive value of different Doppler parameters, such as the umbilical artery pulsatility index (UA PI) in foetuses with growth restriction. Monitoring of the umbilical artery in high risk pregnancies can reduce perinatal mortality and obstetric interventions [5] and optimal timing of delivery is based on changes in the umbilical blood flow [6, 7]. Limited data exist on the use of the middle cerebral artery pulsatility index (MCA PI) as an individual parameter. A recent study by Reis et al. suggests the non-inferiority of MCA PI compared to UA PI in predicting adverse perinatal outcomes [8]. Some studies even suggest that the measurement of MCA PI might be a more sensitive tool in predicting adverse perinatal outcomes in near-term growth restriction [9, 10].

Recently, novel Doppler ratios have been proposed, combining established parameters, such as UA PI, MCA PI, and uterine artery pulsatility index (UtA PI), as well as measurements of amniotic fluid (single deepest pocket, SDP).

An abnormal cerebroplacental Ratio (CPR) has been under discussion as to whether it is associated with an adverse perinatal outcome since the 1980 s [11, 12] and has recently proved to be of diagnostic value, especially in the monitoring of high-risk pregnancies and the diagnosis of a hidden (late) foetal growth restriction [13, 14] A low CPR in women with FGR or SGA is associated with adverse perinatal outcomes [15].

According to Stumpfe et al., a new ratio called the AUCR may help identify pregnancies at high risk of adverse perinatal outcomes. The AUCR combines the UA PI, MCA PI and SDP to provide a comprehensive assessment of foetal health. In a retrospective study of 165 singleton pregnancies with small for gestational age (SGA) foetuses at term, the relationship between UA PI, MCA PI, CPR, and AUCR and adverse perinatal outcomes was shown. AUCR emerged as the strongest predictor of adverse perinatal outcomes, outperforming other parameters in its ability to identify high-risk pregnancies [16].

Currently, there is a notable lack of research on how these Doppler parameters apply to women with preeclampsia, with or without FGR. We hypothesize that the AUCR could not only improve perinatal outcomes in women with SGA foetuses, but also in women with preeclampsia. We, therefore, compare the AUCR as well as other Doppler parameters in pregnancies with and without preeclampsia to optimize risk stratification and the perinatal outcomes of these high-risk pregnancies.

## Method

This study consisted of a single-centre, retrospective analysis. The study was designed according to the Declaration of Helsinki and was approved by the Ethics Committee of the local medical council and the University of Münster (trial number: 2023–074-f-S).

Clinical data were collected from women with and without preeclampsia who underwent an ultrasound examination at the University Hospital Münster between 01/2017 and 12/2023. Women who underwent ultrasound examination for any medical reason, including routine screening, diagnostic evaluation, or monitoring, were eligible for inclusion.

The inclusion criteria consisted of the following: Singleton pregnancies, availability of data for UA PI, MCA PI, SDP, foetal biometry, a maximum interval of 14 days between ultrasound screening and delivery, as well as available data on delivery and perinatal outcome, gestational age between 23 and 42 weeks. Conversely, pregnancies complicated by multiple gestations, congenital anomalies, incomplete data, or stillbirth were excluded from the study.

Gestational age was estimated based on the last menstrual period, with confirmation or adjustment using first-trimester crown-rump length measurements, if necessary.

Ultrasound examinations were performed using PHILIPS EPIQ7 and PHILIPS IU22 (Philips Medical Systems, Andover, MA, USA), with ViewPoint as software for examination documentation (GE HealthCare, Chicago, Illinois, USA). UA PI, MCA PI and SDP, were measured according to ISUOG guidelines [17]. The percentiles for UA PI and MCA PI were calculated based on reference values from the Fetal Medicine Foundation [18].

Estimated foetal weight was calculated using the Hadlock formula, and percentiles were determined based on foetal growth using foetal growth curves from the publication of Marsál et al. [19]. Women with preeclampsia were defined according to the ISSHP guidelines as (de novo) gestational hypertension combined with one or more of the following: Proteinuria, other maternal organ dysfunction, or uteroplacental dysfunction [2].

Foetal growth restriction was defined according to the ISUOG guidelines [20].

We calculated the CPR and AUCR for each participant. AUCR was defined as previously described by Stumpfe et al. [16]: SDP/(UA-PI/MCA-PI). The formula by Stumpfe et al. was not modified and the SDP was recorded in centimetres. Reference percentiles for CPR were derived from the Fetal Medicine Foundation dataset [18].

Data on the patients’ characteristics and the perinatal outcomes were collected from the electronic patient files. When regarding the perinatal outcome, the following parameters were analysed:

Birth weight < 10th percentile, preterm birth, need for operative intervention (vaginal operative or secondary caesarean section), as well as composite adverse perinatal outcomes (CAPOs), based on the work of Stumpfe et al. [16]. The CAPO definition by Stumpfe et al. was adopted without modification and was defined as the presence of at least one of the following adverse neonatal outcomes: a 5 min APGAR score < 7, umbilical cord arterial pH < 7.1, neonatal intensive care unit (NICU) admission [16].

## Statistical analysis

All statistical analyses were performed using SPSS (versions 29 and 31, IBM Corporation, Armonk, NY, USA).

All continuous data were expressed as medians with 25th and 75th interquartile ranges, while categorical variables were presented as numbers and percentages.

Since physiological changes in the resistance of the umbilical artery and middle cerebral artery occur throughout pregnancy, gestational age-adjusted percentiles from the Fetal Medicine Foundation were used for UA PI, MCA PI, and CPR to account for these developmental changes.

The group comparisons of the variables were performed using Fisher’s exact test or Mann–Whitney U test, respectively.

ROC analyses were conducted to assess the predictive power of various Doppler parameters (UA PI, MCA PI, CPR, and AUCR) on neonatal outcomes. The values of the CPR and AUCR, for which lower values are associated with an increased risk of adverse outcome, were multiplied by −1 prior to analysis. This transformation aligned the direction of all Doppler parameters, such that higher values consistently indicated a higher risk of adverse outcome, thereby allowing direct comparison of the corresponding area under the curve (AUC). The AUC for each parameter was compared using DeLong’s test for pairwise comparisons.

A p-value of < 0.05 was considered significant.

## Results:

Based on our inclusion and exclusion criteria, 151 pregnancies were included in the final analysis: 54 women with preeclampsia and 97 healthy controls.

Women with preeclampsia in our study population had a significantly higher body mass index (27.8 kg/m^2^ vs. 24.2 kg/m^2^, p-value = 0.005) with a higher rate of overweight and obesity (65% vs. 42.3%, p-value = 0.008). In contrast, the groups did not differ significantly in age (32 years vs. 33 years, p-value = 0.78) (Table 1).

**Table 1:** Comparative analysis of demographic and pregnancy-related data between preeclampsia and control group

	Preeclampsia n = 54	Healthy controls n = 97	p-value
Age (years), median (range)	32 (19–42)	33 (18–44)	0.78
BMI (kg/m^2^), median (range) BMI ≥ 25 kg/m^2^, number (%) BMI ≥ 30 kg/m^2^, number (%)	27.75 (17.22–44.80) 35 (65.0%) 19 (35.0%)	24.24 (16.42–38.89) 41 (42.3%) 19 (19.6%)	0.005* 0.008* 0.034*
**Gravida**			
Primigravida (G1), number (%)	27 (50%)	30 (30.93%)	0.02*
Multigravida (≥ G2), number (%)	27 (50%)	67 (69.07%)	0.02*
**Parity**			
Primipara (P0), number (%)	21 (38.89%)	39 (40.21%)	1
Multipara (≥ P1), number (%)	33 (61.11%)	58 (59.79%)	1

BMI: body mass index

^*^ p < 0.05, statistically significant

Mean gestational age at Doppler measurement was significantly lower in patients with preeclampsia (31.74 weeks vs. 37.86 weeks p-value < 0.001). The time interval between Doppler measurement and delivery was significantly shorter in women with preeclampsia (4.50 days vs. 6.92 days, p-value = 0.001).

FGR occurred in 33 (61%) of pregnancies in women with preeclampsia, of which 23 cases of early FGR were detected. Five foetuses (9%) had an estimated foetal weight below the 10th percentile without meeting the criteria for FGR.

Gestational age at delivery among women with preeclampsia was significantly lower than in the healthy controls (median of 34 weeks versus 39 weeks, p-value < 0.001). Premature labour was more common in women with preeclampsia (85% vs. 3%, p-value < 0.001), NICU admission was more frequent (89% vs. 21%, p-value < 0.001) and the rate of primary caesarean section higher (74% vs. 28%, p-value < 0.001). Infants born to women with preeclampsia were more likely to have a birth weight below the 10th percentile, indicating low birth weight (33% vs. 12%, p-value = 0.001) (Table 2).

**Table 2:** Comparative analysis of perinatal outcome and obstetric outcome data between preeclampsia and control group

	Preeclampsia n = 54	Healthy controls n = 97	p-value
Gestational age at delivery, median (range)	33.71 (23.86–39.00)	39 (36.57–41.57)	< 0.001*
**Preterm delivery, number (%)** Late/moderate preterm, number (%) Early preterm, number (%) Extremely preterm, number (%)	46 (85%) 21 (39%) 21 (39%) 4 (7%)	3 (3%) 3 (3%) 0 0	< 0.001* < 0.001* < 0.001* < 0.001*
Birth weight (g), median (range)	1523 (350–3915)	3280 (2335–4500)	< 0.001*
Birth weight Percentile < 10, number (%)	18 (33%)	12 (12%)	0.001*
**Mode of delivery** Primary C-Section, number (%) Spontaneous, number (%) Vaginal operative, number (%) Secondary Caesarean Section, number (%)	40 (74%) 6 (11%) 1 (2%) 7 (13%)	27 (28%) 41 (42%) 9 (9%) 19 (20%)	< 0.001* < 0.001* 0.97 0.37
**Unfavourable outcome** CAPO number (%) • NICU admission, number (%) • Umbilical cord pH < 7.10 (%) • APGAR 5 < 7 (%) Umbilical cord pH median (range) APGAR 5 median (range)	48 (89%) 48 (89%) 1 (2%) 3 (6%) 7.30 (6.9–8.0) 9 (2–10)	20 (21%) 20 (21%) 1 (1%) 4 (4%) 7.26 (7.02–7.45) 10 (4–10)	< 0.001* < 0.001* 1 0.701 0.007* < 0.001*
**Foetal growth** FGR number (%) SGA number (%) ≥ 10.−90. Percentile, number (%)	33 (61%) 5 (9%) 16 (30%)	0 0 97 (100%)	< 0.001* < 0.001* < 0.001*

Abbreviations: Preterm delivery: delivery at < 37 + 0 weeks of gestation, late/moderate preterm delivery: delivery at 32 + 0 to 36 + 6 weeks of gestation, very preterm (28 + 0 to less than 31 + 6 weeks of gestation), extremely preterm (delivery at < 28 + 0 weeks of gestation), NICU: neonatal intensive care unit, CAPO: composite adverse perinatal outcome, FGR: foetal growth restriction, SGA: small for gestational age

^*^ p < 0.05, statistically significant

In women with preeclampsia, median values for UA PI were significantly higher (1.24 vs. 0.89, p < 0.001), whereas CPR was significantly lower (1.21 vs. 1.76, p < 0.001) compared to the control group. Although the absolute values of MCA PI did not differ significantly between groups, analysis using percentiles revealed significantly lower values in women with preeclampsia (see Table 3). Median values for AUCR were significantly lower in the preeclampsia group (4.92 vs. 7.82, p < 0.001). A subgroup analysis of the preeclampsia group revealed no significant difference in the AUCR regarding pregnancies with or without FGR (4.5 vs. 5.04, p = 0.40).

**Table 3:** Comparative analysis of Doppler parameters between preeclampsia and control group

	Preeclampsia	Healthy controls	p-value
Doppler AU PI, median, (IQR), range *Minimum—Maximum*	1.24 (0.4), 2.6 *0.72–3.30*	0.89 (0.20), 0.90 *0.62–1.52*	< 0.001*
Doppler AU PI percentile, median, (IQR), range *Minimum—Maximum*	92.50 (33.50), 88 *12–100*	62.00 (42.5), 95 *5–100*	< 0.001*
Doppler MCA PI, median, (IQR), range *Minimum—Maximum*	1.53 (0.57), 1.63 *0.75–2.38*	1.57 (0.47), 1.58 *0.80–2.38*	0.858
Doppler MCA percentile, median, (IQR), range *Minimum—Maximum*	9.00 (51.25), 98 *1–99*	45.00 (74), 99 *1–100*	< 0.001*
CPR, median, (IQR), range *Minimum—Maximum*	1.21 (0.74), 2.11 *0.48–2.58*	1.76 (0.61), 2.16 *0.88–3.04*	< 0.001*
CPR percentile, median, (IQR), range *Minimum—Maximum*	2.00 (17.75), 90 *1–91*	41.00 (56), 99 *1–100*	< 0.001*
SDP in cm, median, (IQR), range *Minimum—Maximum*	4.37 (2.05), 6.29 *1.01–7.30*	4.54 (2.05), 9.44 *1.36–10.80*	0.57
AUCR, median, (IQR), range *Minimum—Maximum*	4.92 (3.58), 16.43 *1.13–17.57*	7.83 (4.64), 24.48 *1.81–26.29*	< 0.001*

Data presented as median and interquartile range

Abbreviations: IQR: Interquartile range, UA PI: umbilical artery pulsatility index, MCA PI: middle cerebral artery pulsatility index, CPR: cerebro-placental ratio, SDP: single deepest pocket, cm: centimetres, AUCR: amniotic-umbilical-to-cerebral ratio

^*^ p < 0.05, statistically significant

Pathological UA PI, MCA PI, and CPR were significantly associated with adverse perinatal outcome (odds ratio (OR) for CAPO: OR (UA PI) = 12, OR (MCA PI) = 6 and OR (CPR) = 13), preterm labour: OR (UA PI) = 14, OR (MCA PI) = 4 and OR (CPR) = 7 and operative intervention during labour OR (UA PI) = 5, OR (MCA PI) = 3 and OR (CPR) = 7).

**Fig. 1:**
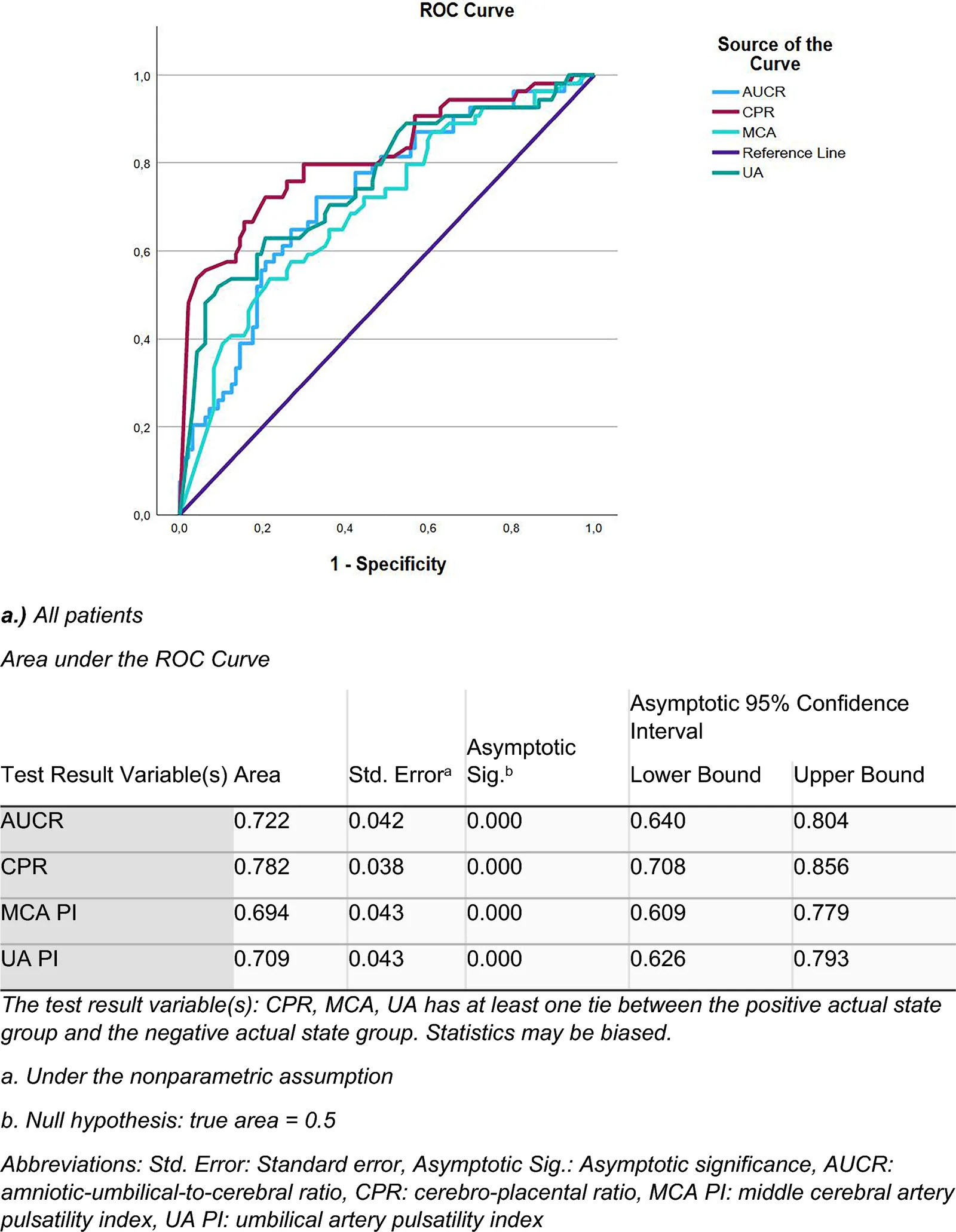

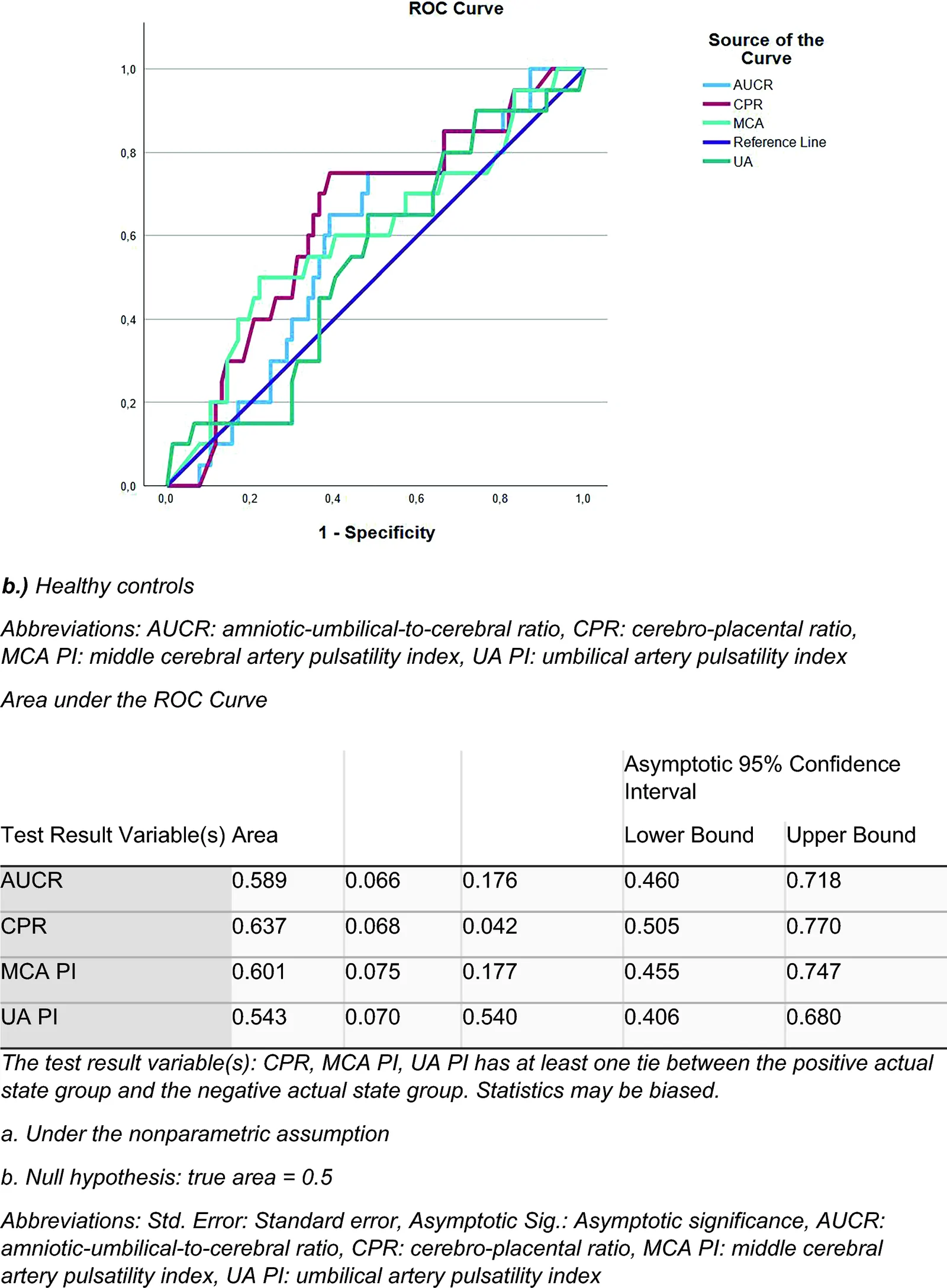

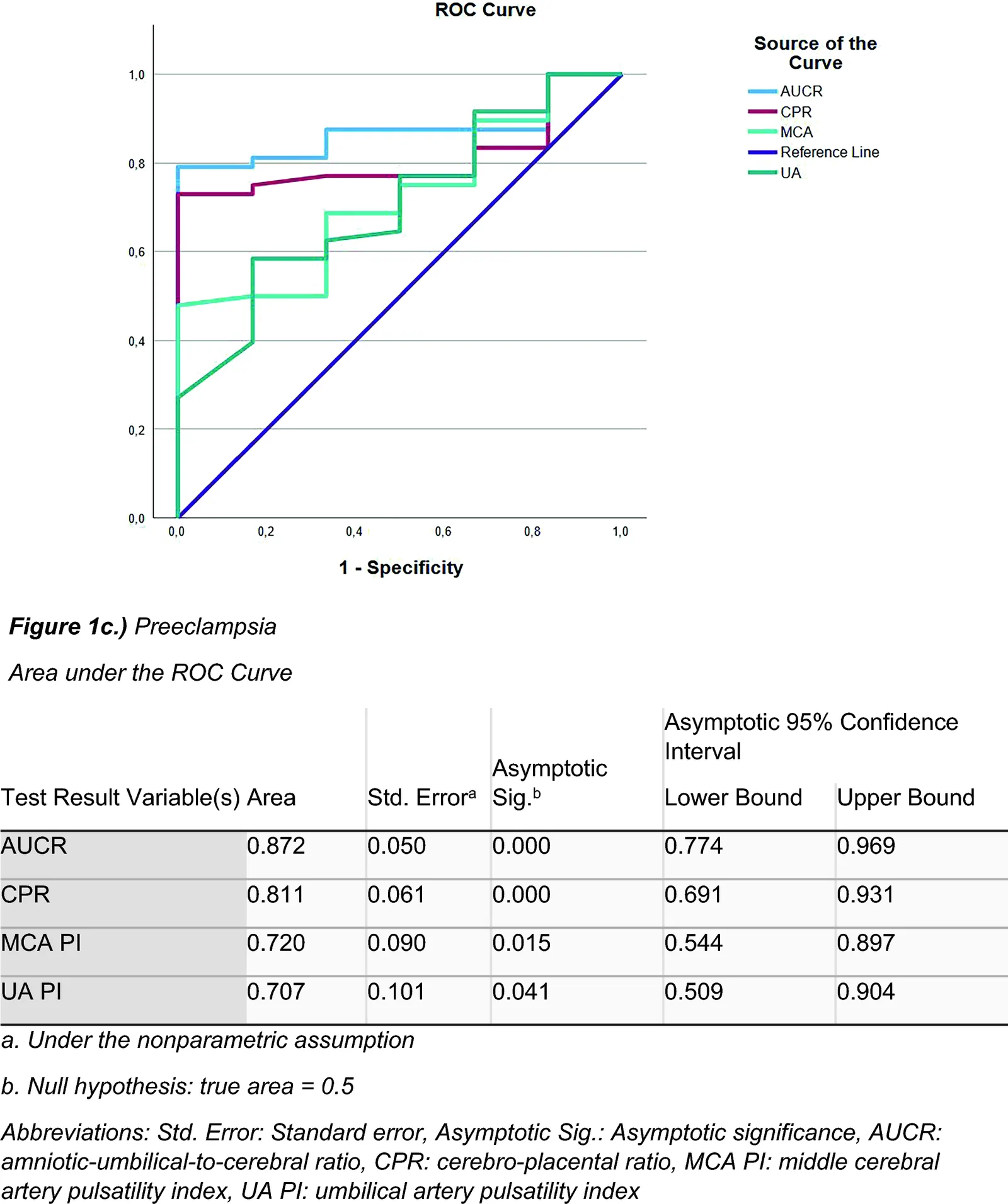
Abbreviations: ROC curves for different Doppler parameters predicting CAPO. AUCR: amniotic-umbilical-to-cerebral ratio, CPR: cerebroplacental ratio, MCA PI: middle cerebral artery pulsatility index

The results of the ROC analysis for UA PI, MCA PI, CPR, and AUCR are presented in Fig. 1, stratified by healthy controls, women with preeclampsia, and the entire study population.

Among women with preeclampsia, the AUCR exhibits the largest AUC for predicting CAPO (AUC: 0.872), followed by CPR (AUC: 0.811), MCA PI (AUC: 0.72), and UA PI (AUC 0.707). Differences in AUC between different Doppler parameters did not reach statistical significance (Table 4).

**Table 4:** Differences in area under the curve (AUC) among Doppler parameters in patients with preeclampsia

	p-value	95% CI lower bound	95% CI upper bound
AUCR vs. CPR	0.144	−0.021	0.142
AUCR vs. MCA PI	0.084	−0.020	0.323
AUCR vs. UA PI	0.097	−0.030	0.360
CPR vs. MCA PI	0.234	−0.058	0.239
CPR vs. UA PI	0.308	−0.096	0.304
MCA PI vs. UA PI	0.934	−0.314	0.342

CI: confidence interval, AUCR: amniotic-umbilical-to-cerebral ratio, CPR: cerebro-placental ratio, MCA PI: middle cerebral artery pulsatility index, UA PI: umbilical artery pulsatility index

Comparison of AUC between groups showed that AUCR had a significantly higher AUC in women with preeclampsia than in healthy controls (0.872 vs. 0.589, p < 0.001). The AUC for CPR, MCA PI and UA PI were also higher in women with preeclampsia; however, these differences were not statistically significant (CPR: 0.811 vs. 0.637, p = 0.058, MCA PI: 0.720 vs. 0.601, p = 0.306, UA PI: 0.707 vs. 0.543, p = 0.182).

## Discussion

Our results indicate that a combined ratio of established Doppler parameters, including UA PI and MCA PI, along with the SDP as part of the AUCR, might enhance the identification of pregnancies at high risk for adverse perinatal outcomes in women with preeclampsia.

To our knowledge, only one study has specifically examined the value of AUCR in women with preeclampsia. Karabay et al. noted that implementing AUCR could help identify the risk of adverse perinatal outcomes in pregnancies complicated by preeclampsia, as the underlying pathomechanism of placental insufficiency is shared in both preeclampsia and FGR [21]. Although FGR is common in women with preeclampsia, the aforementioned study excluded foetuses with suspected growth restriction. The results of our study demonstrated significantly lower AUCR values in preeclamptic pregnancies, regardless of FGR status, compared to healthy controls. In addition, subgroup analysis within the preeclampsia cohort revealed no significant difference in AUCR between pregnancies with and without FGR. Taken together, these findings do not support the assumption that the observed performance of AUCR in patients with preeclampsia is solely driven by the high prevalence of FGR within this cohort. This interpretation is consistent with the findings reported by Karaby et al. [21].

Especially in women with preeclampsia, the utility of novel Doppler ratios, both with and without FGR, remains understudied.

These findings also align with previous research on foetuses with growth restriction [21, 22], which demonstrated that AUCR can offer enhanced prognostic value compared to individual Doppler measurements, especially in patients with a high risk of adverse perinatal outcomes. Both studies showed an association between adverse perinatal outcomes and late FGR.

Preeclampsia, particularly early onset preeclampsia, is often linked to FGR due to a shared underlying factor: placental dysfunction [23]. While the exact mechanisms behind preeclampsia are not yet fully understood, research suggests that impaired placental function plays a crucial role in the development of this condition [24]. Consequently, it is not surprising that a diagnostic tool, such as AUCR, which can improve prenatal care in women with suspected growth restriction, yields similar results in women with preeclampsia.

To date, no standardized cut-offs for the interpretation of AUCR exist.

According to international and national guidelines, measurement of UA PI in the monitoring of growth-restricted foetuses is the standard of care [1, 20]. A decreased CPR (< 1) in growth-restricted foetuses is associated with increased morbidity in newborns [25] and worse neurological outcomes in early childhood [26].

Limited data exist for women with preeclampsia and the implication of CPR beyond growth restriction in preeclampsia remains speculative. A case–control study with only 50 participants showed increased neonatal mortality in women with preeclampsia when CPR was < 1 [27]. In our study, CPR was associated with adverse perinatal outcomes.

Oligohydramnios is a common finding in women with FGR [28]. Although there appears to be an association between low birth weight and oligohydramnios, oligohydramnios alone is a poor predictor of adverse perinatal outcomes in women with FGR [28, 29]. Foetuses with growth below the 3rd percentile with oligohydramnios have a worse outcome compared to those growth-restricted foetuses below the 3rd percentile with a normal amount of amniotic fluid.

The impact of oligohydramnios on adverse perinatal outcomes in pregnancies complicated by preeclampsia remains unclear. Although we did not detect a strong direct link between oligohydramnios and poor perinatal outcomes, our findings suggest that the AUCR, which incorporates the SDP measurement of amniotic fluid, is a relevant factor in assessing perinatal risk and should be taken into consideration when monitoring these pregnancies.

## Strengths and limitations

Our study's retrospective design may introduce biases and limit the accuracy of our findings. The small sample size of our study cohort may not be representative of the larger population, which could affect the validity of our results. A further subdivision of the preeclampsia cohort into subgroups, apart from the FGR analysis, was not performed because of the small sample size (e.g., based on disease severity or on the therapeutic interventions that were initiated). Therefore, confounding factors cannot be ruled out. Differences in AUC between the analysed Doppler parameters did not reach statistical significance, which limits reliability. This could be explained by the small cohort.

AUCR demonstrates high predictive ability, yet its performance is comparable to that of established Doppler indices in this cohort. When interpreting the ROC comparison between the preeclampsia group and the controls, it should be acknowledged that Doppler measurements in the preeclampsia group were obtained at a significantly lower gestational age. This is particularly relevant because established Doppler indices, including MCA PI and UA PI, are known to vary with gestational age [30]. It is, therefore, reasonable to assume that the AUCR may likewise be influenced by gestational age, although gestational age-specific reference ranges or percentiles for AUCR are currently unavailable. Consequently, the observed differences in diagnostic performance between the two groups may, at least in part, reflect differences in gestational age assessment rather than solely disease related effects. Accordingly, the results of the ROC comparison should be interpreted with caution and considered exploratory in nature. Despite these limitations, the AUCR exhibited a strong predictive ability in the study group, as reflected by an AUC of 0.87. Nevertheless, owning to the relatively small sample size, these findings should be considered preliminary and warrant validation in larger, prospective trials.

While a gestational age-matched control group would improve the comparability of the study and control group, Doppler and amniotic fluid measurements were designed to be collected shortly before delivery, as described by Stumpfe and colleagues [16]. As a result, establishing an appropriate healthy control group without relevant diseases or risk factors is challenging, since AUCR measurements would need to be performed shortly before birth and followed by preterm delivery at a gestational age similar to that observed on the preeclampsia group. This limits the feasibility of recruiting a truly comparable gestational age-matched control cohort.

Moreover, we did not have access to long-term outcome data, which would have provided valuable insights into the prognostic value of our findings.

A key strength of this study is that, to our knowledge, it is the first to focus specifically on the implementation of AUCR as a novel ratio in pregnancies complicated by preeclampsia, with or without FGR, in comparison with healthy controls. The study's dataset comprised 54 women with preeclampsia and 97 healthy controls, providing a substantial sample size that enhances the reliability of the findings. This unique focus enables a meaningful exploration of the potential benefits and applications of AUCR in this specific clinical context.

## Conclusion

In this retrospective cohort, AUCR demonstrated good discriminative ability for adverse perinatal outcomes (AUC = 0.87) in preeclamptic patients, but did not outperform established Doppler indices. Given the limited sample size and lack of a validated cut off, AUCR should be regarded as a promising adjunct pending confirmation in larger, prospective studies.

## Data Availability

Data supporting the results of the study are included in the manuscript. Raw data is available upon request.
